# Synergistic effects of Buddhist five precepts and death contemplation on inner strengths and mental health in elderly Thai meditators

**DOI:** 10.1186/s40359-025-03679-9

**Published:** 2025-12-08

**Authors:** Alla Glushich, Justin DeMaranville, Tinakon Wongpakaran, Danny Wedding, Nahathai Wongpakaran

**Affiliations:** 1 Mental Health Program, Multidisciplinary and Interdisciplinary School, Chiang Mai University, Chiang Mai, 50200 Thailand; 2 Department of Psychiatry, Faculty of Medicine, Chiang Mai University, Chiang Mai, 50200 Thailand; 3 Department of Clinical and Humanistic Psychology, Saybrook University, Pasadena, CA 91103 USA; 4 Missouri Institute of Mental Health, University of Missouri-Saint Louis, St. Louis, MO 63121 USA

**Keywords:** Precepts, Death meditation, Mental health, Older adults

## Abstract

**Background:**

This study investigated pāramīs-based inner strengths and mental health outcomes among elderly Thai Buddhist meditators, with a particular focus on the combined effects of strict ethical adherence to the Five Precepts (abstaining from killing, stealing, sexual misconduct, lying, and intoxicants) and varying level of death contemplation practice because this combination is a hallmark of serious meditation practice among those aspiring to attain nirvana, the ultimate spiritual goal in Buddhism.

**Methods:**

A cross-sectional sample of 332 participants aged 60 and above was recruited from temples and meditation centers in Northern Thailand. Using validated self-report measures, we assessed the frequency of observance of the Five Precepts, engagement in maranasati meditation, the ten pāramī virtues including generosity, morality, mindfulness/meditation, wisdom, perseverance, patience, truthfulness, determination, loving-kindness, and equanimity, and a range of mental health indicators, including depression, anxiety, aggression, well-being, gratitude, life satisfaction, and self-esteem.

**Results:**

Dedicated practitioners, who had perfect adherence to the Five precepts and engagement in death contemplation practice, demonstrated significantly higher levels of pāramī-based inner strengths, as well as greater well-being, gratitude, and life satisfaction. They also showed lower levels of anxiety, depression, and aggression than less dedicated peers. However, no significant differences in gratitude were observed between groups, suggesting that gratitude may not primarily depend on the combination of ethical and contemplative practices.

**Conclusion:**

These findings highlight the unique and synergistic benefits of combining rigorous ethical conduct with contemplation of impermanence in fostering resilience and psychological flourishing among older adults. The results underscore the enduring significance of Buddhist principles for mental health and spiritual well-being in contemporary aging populations, while also clarifying that some positive psychological traits, such as gratitude, may be influenced by factors beyond formal Buddhist practice. Future longitudinal and cross-cultural studies are warranted to clarify causality and generalizability.

## Introduction

Buddhist practice provides a comprehensive framework for the pursuit of spiritual development and psychological well-being. Central to this tradition is the Noble Eightfold Path, classically grouped into the Threefold Training: ethical conduct (Sīla), meditation (Samādhi), and wisdom (Paññā). These practices aim to help practitioners reduce suffering (dukkha) by cultivating virtue, mental discipline, and insight into impermanence (anicca) and non-self (anatta) [1, 2].

Concepts of ‘spirituality’ and ‘religiosity’ often overlap in empirical research. However, the former generally refers to internal processes, such as the search for personal meaning, and the latter to more external, community-oriented practices [3]. In Buddhism, religious development typically refers to an individual’s external engagement with the tradition, such as participation in rituals, communal observance of the precepts, and adherence to established cultural or institutional norms [4]. Such practices both reinforce a shared framework of values and connect individuals with the wider Buddhist community (Sangha). In contrast, spiritual development emphasizes the internal, experiential dimension of practice: the cultivation of personal insight and wisdom (paññā), often through transformative practices like meditation, culminating in direct realization of key Buddhist teachings such as impermanence and non-self.

The Five Precepts, which include abstaining from killing, stealing, sexual misconduct, lying, and intoxicants, anchor the ethical dimension of practice, supporting mindful living and harmonious relations [4, 5]. Meditation, including the advanced practice of death contemplation (maranasati), fosters present-moment awareness and deep acceptance of impermanence [6–8]. The cultivation of wisdom through practices such as maranasati is also emphasized by advanced practitioners, as a direct engagement with mortality. Wisdom emerges as practitioners increasingly recognize that suffering arises from attachment and aversion, and that all phenomena are transient [4, 9].

Despite the central importance of these pillars within traditional Buddhism, empirical research has predominantly focused on the mental health benefits of meditation [10, 11], often neglecting the roles of ethical conduct and wisdom, especially in naturalistic settings. Whereas meditation is an active mental process which may result in affect changes a person can immediately feel benefit from, precept observances involve vigilance, discipline, and refrainment from common behaviours such as drinking alcohol or killing mosquitoes. Research on a preference for high vs. low arousal states between Western and Eastern cultures may also explain the neglect of precept observances as a worthwhile Buddhist practice to adopt [12]. However, even Thai Buddhists struggle to keep the precepts in a modern, consumerist culture [13], another influence that may contribute to the low proportion of Thai people who are precept holders. Notably, only about 9–22% of Thai Buddhists are found to be fully observing the Five Precepts, and this subset is typically comprised of highly dedicated practitioners striving toward higher spiritual milestones [14–17].

Observing the Five Precepts has been linked with greater psychological strengths and happiness [10, 18], higher life satisfaction [19], and lower rates of depression—especially among older adults [14]. The Five Precepts are also associated with reduced risky behaviors among youth [20] and offer protection against relational challenges such as insecure attachment and depression [21, 22]. Conversely, breaches of the Precepts are correlated with increased risk for various mental health issues, including violence and substance abuse [23–36]. The mental health benefits and protective nature of the precepts against risky behaviours and associated outcomes are why precepts are adopted first when entering the temple life or a meditation retreat in Thailand [37]. Refraining from morally risky behaviours prepares the mind to be focused during meditation.

Among the many practices central to the Buddhist path, ethical conduct (Sīla) and contemplative wisdom (Paññā) work together to support personal transformation. While the Five Precepts lay the foundation for moral integrity and harmonious living, more advanced practices—such as death contemplation, or maranasati—invite practitioners to face life’s impermanence directly. Traditionally, maranasati is regarded as a practice for experienced meditators, challenging individuals to reflect on the certainty and unpredictability of death. Far from encouraging passivity or fear, this reflection prepares the mind to embrace each moment and strengthens motivation for virtuous conduct in daily life [38, 39]. The cultivation of such awareness, especially in cultures like Thailand where death contemplation is valued, can foster equanimity and resilience. Experimental and clinical research increasingly shows that rather than inducing anxiety, mindful engagement with mortality can reduce fear, promote gratitude, and enhance well-being and social connectedness [40–46]. In the Buddhist context, this acceptance of impermanence and mortality forms part of a broader constellation of character strengths—such as truthfulness, perseverance, wisdom, generosity, patience, determination, and loving-kindness—that have themselves been linked to psychological resilience and lower rates of depression [47, 48]. Yet, despite its central role in Buddhist teachings, maranasati has received much less empirical attention than other forms of meditation, and even less is known about how it interacts with foundational practices like the Five Precepts. Existing studies of ethical conduct often focus narrowly on how many laypeople maintain precept observance, rather than investigating the potential psychological and developmental benefits it may confer. Similarly, research on maranasati has tended to emphasize its association with death anxiety, overlooking its capacity to cultivate acceptance or support positive adaptation [49]. Few investigations have examined how spiritual engagement evolves over the lifespan—or whether combining long-term ethical commitment with regular death contemplation offers unique psychological advantages for older adults [50, 51].

To address these gaps, the present study investigates psychological outcomes among elderly Buddhist practitioners with varying profiles of religious and spiritual engagement, as defined by their adherence to the Five Precepts and practice of maranasati meditation. Participants were classified into four groups according to their levels of precept adherence (high or low) and frequency of maranasati practice (high or low). Of particular interest are the “dedicated practitioners,” defined as those who show both high adherence to the Five Precepts and regular engagement in maranasati.

By comparing these groups on a range of psychological measures—including inner strengths (pāramīs), aggression, depression, anxiety, well-being, life satisfaction, self-esteem, and gratitude—this factorial design allows us to explore the unique and combined effects of Buddhist ethical and contemplative practices. We hypothesized that dedicated practitioners would demonstrate the most favorable psychological profiles, exhibiting greater well-being, life satisfaction, self-esteem, gratitude, and inner strengths, and lower levels of anxiety, depression, and aggression than all other groups.

## Methods

### Study design

This cross-sectional study examined the psychological and spiritual outcomes associated with advanced Buddhist practice among elderly Thai meditators. Data were collected between August and October 2024.

### Participants

A total of 332 Buddhist meditators, aged 60 years or older (per the Thai legal definition of “elderly”), were recruited from temples and meditation centers in Northern Thailand.

Contact information was obtained online from publicly available resources. A Thai-speaking research assistant then contacted temples and meditation centers to inquire about the possibility of data collection, providing information about the study’s focus on death contemplation training. This specific recruitment strategy was used to ensure the inclusion of genuinely dedicated meditators rather than the general senior population. Although forty meditation methods are documented among Thai Buddhists, this study focused exclusively on practitioners who reported prior experience with death-contemplation (maranasati) meditation, regardless of their primary meditation method. During initial contact with potential data collection sites, the assistant confirmed that the sites’ practitioners were familiar with death contemplation practice, though not necessarily currently practicing it.

If the administrators approved data collection and provided appropriate space for data collection, they were provided with the necessary documents (including a permission letter for data collection confirming ethical approval, a participant information sheet, an informed consent form, a compensation receipt, and a questionnaire). On the agreed-upon date, data were collected at a designated area within a temple or meditation center, under the guidance of Thai-speaking research assistants. Information about the study was also shared on social media (including Facebook and Instagram), enabling online participation via a Microsoft Forms survey.

All participants provided informed consent, and the study protocol was approved by the Research Ethics Committee of the Faculty of Medicine, Chiang Mai University (ID: PSY-2567-0372) on August 9, 2024.

### Instruments

Sociodemographic data—age, gender, education, and employment status—were collected. Instruments measuring death contemplation, precept adherence, paramī-based inner strengths, anxiety, depression, aggression, well-being, life satisfaction, self-esteem, and gratitude are described below.

#### Death contemplation

The Modified Meditation Evaluation Questionnaire (MMEQ) was adapted to assess the frequency of death contemplation meditation (maranasati) per month and per day [52]. Frequency is rated on a five-point scale: 0 (never), 1 (hardly ever), 2 (sometimes), 3 (quite often), and 4 (very often).

#### Precept adherence

An item from the inner Strengths-Based Inventory was used to assess precept adherence [49]. Item 5 allows five response options: “I never thought of following the five precepts.” (score of 1), “It is difficult for me to follow all the Five Precepts.” (score of 2), “I intend to follow the Five Precepts, but sometimes I cannot.” (score of 3), “I can follow the Five Precepts but break them once in a while.” (score of 4), and “I always follow the Five Precepts. As far back as I can remember, I have never violated them.” (score of 5, perfect adherence).

#### Inner strengths

Pāramī-based inner strengths were assessed using the Inner Strengths-Based Inventory, assessing generosity, morality, mindfulness/meditation, wisdom, perseverance, patience, truthfulness, determination, loving-kindness, and equanimity [49]. Each item was rated on a five-point scale, reflecting the perceived level of the respective strength. Total scores were calculated by summing responses across items, excluding the morality item (based on the Five Precepts). The higher scores reflect greater strengths. The iSBI (item 5 excluded) demonstrated strong internal consistency, with a Cronbach’s alpha of 0.885.

#### Anxiety

Anxiety was assessed using the anxiety subscale of the Outcome Inventory (OI-21) [53]. The subscale consists of six items, scored on a 5-point Likert scale (0 = “never” to 4 = “almost always”), with higher scores indicating greater symptoms. Total score range is 0–24.

#### Depression

Depression was assessed using the depression subscale of the Outcome Inventory (OI-21) [53]. The subscale consists of five items, scored on a five-point Likert scale (0 = “never” to 4 = “almost always”), where higher scores reflect greater symptoms. Total score range is 0–20.

#### Aggression

Aggression Subscale of the Zuckerman-Kuhlman-Aluja Personality Questionnaire (ZKA-20) [54]. The subscale consists of four items, scored on a four-point Likert scale (scores range from 4 to 16), with higher scores indicating more pronounced aggressive traits. Cronbach’s alpha in the current sample was 0.83.

#### Well-being

The Thai version of the WHO-5 Well-Being Index was used to evaluate general well-being [55]. Scores range from 0 to 25 and are multiplied by 4 for a percentage score. A score below 50 represents poor mental well-being. The higher scores reflect a greater state of mental well-being. Cronbach’s alpha in this sample was 0.925.

#### Life satisfaction

The Multidimensional Life Satisfaction Assessment (MLSA) was used to assess life satisfaction. This 8-item instrument was developed specifically for use in older adult Thais [56]. Internal consistency in the current sample was high, with Cronbach’s alpha being 0.908.

#### Gratitude

The Gratitude Inventory (GI-6) was used to assess gratitude [57]. This six-item measure was developed with roots in Thai culture. Responses are rated on a 5-point Likert scale, with the total score ranging from 6 to 30. Cronbach’s alpha was 0.940.

#### Self-esteem

The Rosenberg Self-Esteem Scale Thai Revised (RSES-TR) was used to measure self-esteem [58]. The scale consists of 10 items, and responses are rated on a four-point Likert scale, with the total score ranging from 0 to 30. A higher score represents higher self-esteem. Among the current sample, Cronbach’s alpha was 0.786.

### Statistical analysis

Descriptive statistics (percentages, means, and standard deviations) were computed for sociodemographic characteristics and independent and dependent variables. Correlation analysis examined associations among continuous measures. Participants were divided into four groups to disentangle the distinct and combined effects of precept adherence and death contemplation, enabling us to assess whether their integration yields greater psychological benefits than either practice alone.

Group 1: those with a low level of precept adherence and a low level of death contemplation practice, Group 2: those with a high level of precept adherence and low level of death contemplation practice, Group 3: those with a low level of precept adherence and a high level of death contemplation practice, and Group 4: those with a high level of precept adherence and a high level of death contemplation practice.

To examine differences in psychological outcomes among practitioner groups, we analyzed covariance (ANCOVA). Four groups were created based on a 2 × 2 design: high vs. low precept adherence and high vs. low frequency of death contemplation practice. Psychological variables (inner strengths, well-being, anxiety, depression, aggression, life satisfaction, self-esteem, and gratitude) served as dependent variables. Age, sex, and other relevant demographic variables were entered as covariates to control for potential confounding effects. This approach allowed for adjusted group comparisons, ensuring that any observed differences in outcomes were not attributable to these covariates. Where a significant main effect of group was found, pairwise post hoc comparisons were performed using the Bonferroni correction to adjust for multiple testing and reduce the risk of Type I error. Adjusted p-values less than 0.05 were considered statistically significant. All statistical analyses were performed using IBM SPSS Statistics, version 26 (IBM Corp., Armonk, NY, USA). Exact p-values are reported, and p-values less than 0.001 are reported as *p* < 0.001.

## Results

A total of 332 elderly Thai Buddhist meditation practitioners participated in this study. The sample was predominantly female (66%), with a mean age of 68.12 years (SD = 6.86). Just under half (49.7%) had attained education beyond high school, and 30.4% were currently employed (see Table 1).

**Table 1:** Demographic information and characteristics (*n* = 332)

Variables	Number	Percent
*Gender*		
Female	219	66
Male	113	34
*Education*		
> High school	165	49.7
≤ High school	167	50.3
*Employment*		
Employed	101	30.4
Unemployed	231	69.6

Descriptive statistics for meditation and mental health measures are presented in Table 2. The average MMEQ score (frequency of death contemplation meditation) was moderate, with 24.7% of participants practicing “quite often” and 25.0% “very often.” Highly dedicated practitioners, defined as those with perfect adherence to the Five Precepts and maranasati practice, comprised 38.9% of the sample. The mean score for pāramīs-based inner strengths was 38.94 (SD = 7.28), with Equanimity and Patience scoring the highest among individual strengths.

**Table 2:** Mental health outcomes, positive mental health factors, and personality instrument scores (*n* = 332)

Variables	*n* (%) or Mean (SD)
MMEQ (0–8) (death meditation frequency)	4.93 (2.12)
Frequency per month	
0. Never	16 (4.8%)
1. Hardly ever	36 (10.8%)
2. Sometimes	138 (41.6%)
3. Quite often	66 (19.9%)
4. Very often	76 (22.9%)
Frequency per day	
0. Never	21 (6.3%)
1. Hardly ever	32 (9.6%)
2. Sometimes	115 (34.6%)
3. Quite often	95 (28.5%)
4. Very often	69 (20.8%)
High level of dedicated practitioners	129 (38.9%)
Pāramīs-based inner strengths sum score (9–45)	34.91 (6.62)
Five Precepts (1–5)	4.03 (0.97)
1. I never thought of following the five precepts.	5(1.5%)
2. It is difficult for me to follow all the Five Precepts.	14(4.2%)
3. I intend to follow the Five Precepts, but sometimes I cannot.	79(23.8%)
4. I can follow the Five Precepts but break them once in a while.	101(30.4%)
5. I always follow the Five Precepts. As far back as I can remember, I have never violated them.	133(40.1%)
Aggression (4–16)	7.86 (3.08)
Depression (0–20)	2.64 (2.78)
Anxiety (0–24)	4.69 (3.68)
Well-being (0–100)	75.99 (18.61)
Life satisfaction (8–32)	25.77 (3.91)
Self-esteem (0–30)	21.48 (4.37)
Gratitude (5–25)	23.22 (3.22)

Note. *SD* Standard deviation, *MMEQ* Modified meditation evaluation questionnaire

Correlation analyses indicated that high-level dedicated practice was positively associated with pāramīs-based inner strengths, gratitude, well-being, life satisfaction, and self-esteem, and negatively correlated with anxiety, depression, and aggression (see Table 3).

**Table 3:** Correlations between covariates and dedicated practitioner, anxiety, depression, aggression, well-being, life satisfaction, and gratitude

		1	2	3	4	5	6	7	8	9	10	11	12	13
1	Age	–												
2	Sex	–0.108*	–											
3	Education	–0.098	0.002	–										
4	Employment	0.181^**^	–0.119*	–0.212**	–									
5	Dedicated practitioner	0.095	–0.038	0.001	–0.024	–								
6	Inner strengths	–0.009	0.023	0.074	–0.159**	0.549^**^	–							
7	Anxiety	–0.007	–0.044	0.014	0.144^**^	–0.239**	–0.396**	–						
8	Depression	–0.016	–0.016	–0.143**	0.152^**^	–0.255**	–0.355**	0.791^**^	–					
9	Aggression	–0.040	0.065	–0.070	–0.002	–0.245**	–0.311**	0.347^**^	0.395^**^	–				
10	Well–being	–0.057	0.020	0.104	–0.029	0.281^**^	0.395^**^	–0.538**	–0.463**	–0.259**	–			
11	Life satisfaction	–0.027	–0.025	0.147^**^	0.071	0.180^**^	0.190^**^	–0.271**	–0.306**	–0.133*	0.350^**^	–		
12	Self–esteem	–0.061	0.038	0.150^**^	–0.099	0.193^**^	0.314^**^	–0.547**	–0.577**	–0.405**	0.478^**^	0.312^**^	–	
13	Gratitude	–0.109*	0.033	0.196^**^	–0.111*	0.110^*^	0.206^**^	–0.390**	–0.408**	–0.216**	0.377^**^	0.262^**^	0.319^**^	–

Note: *p < 0.05, **p < 0.01

In the ANOVA, the number of the participants according to the groups were as follows.

Group 1: those with a low level of precept adherence (scores 0–4) and a low level of death contemplation practice (Low P and Low DC, *n* = 149), Group 2: those with a high level of precept adherence (score 5) and a low level of death contemplation practice (scores 0–5) (High P and Low DC, *n* = 52), Group 3: those with a low level of precept adherence (scores 0–4) and a high level of death contemplation practice (scores 6–8) (Low P and High DC, *n* = 49), and Group 4: those with a high level of precept adherence (score 5) and a high level of death contemplation practice (6–8)(High P and High DC, *n* = 81).

Table 4 presents ANCOVA results comparing psychological outcomes among four groups defined by levels of precept adherence (high/low) and frequency of death contemplation practice (high/low), controlling for covariates. Significant differences were found for most variables, including inner strengths, anxiety, depression, aggression, well-being, life satisfaction, and self-esteem. In all of these domains, the High P, High DC group had the most favorable scores—showing significantly greater inner strengths, well-being, life satisfaction, and self-esteem, as well as lower levels of anxiety, depression, and aggression compared to the Low P, Low DC, and other groups. However, there were no significant group differences in gratitude. These results highlight the combined benefit of both high precept adherence and regular death contemplation practice for various aspects of psychological well-being.

**Table 4:** ANCOVA results of differences between different levels of practitioners, controlling for covariates

	Practitioner	Mean	SD	F(df1,df2), *p*-value
Inner Strengths	Low P, Low DC	35.01	6.04	(3,323) = 52.230, *p* < 0.001 High P, High DC > Low P, Low DC (*p* < 0.001) High P, High DC > Low P, High DC (*p* < 0.001)
	High P, Low DC	43.17	5.48	
	Low P, High DC	36.55	6.03	
	High P, High DC	45.14	4.99	
Anxiety	Low P, Low DC	5.58	3.77	(3,323) = 7.590, *p* < 0.001 High P, High DC > Low P, Low DC (*p* < 0.001)
	High P, Low DC	3.90	3.66	
	Low P, High DC	4.61	3.37	
	High P, High DC	3.51	3.25	
Depression	Low P, Low DC	3.39	2.87	(3,323) = 8.904, *p* < 0.001 High P, High DC > Low P, Low DC (*p* < 0.001)
	High P, Low DC	1.90	2.59	
	Low P, High DC	2.63	2.77	
	High P, High DC	1.69	2.27	
Aggression	Low P, Low DC	8.827	2.83	(3,323) = 10.435, *p* < 0.001 High P, High DC > Low P, Low DC (*p* < 0.001)
	High P, Low DC	7.632	3.51	
	Low P, High DC	7.492	2.82	
	High P, High DC	6.602	2.82	
Well-being	Low P, Low DC	17.72	4.68	(3,323) = 11.606, *p* < 0.001 High P, High DC > Low P, Low DC (*p* < 0.001) High P, High DC > Low P, High DC (*p* = 0.026)
	High P, Low DC	19.12	4.58	
	Low P, High DC	18.98	4.49	
	High P, High DC	21.33	3.84	
Life satisfaction	Low P, Low DC	25.11	3.41	(3,323) = 4.064, *p* = 0.02 High P, High DC > Low P, Low DC (*p* < 0.01)
	High P, Low DC	25.92	4.04	
	Low P, High DC	25.59	4.10	
	High P, High DC	27.00	4.34	
Self-esteem	Low P, Low DC	30.46	3.95	(3,323) = 5.754, *p* < 0.001 High P, High DC > Low P, Low DC (*p* = 0.002) High P, Low > Low P, Low DC (*p* = 0.033)
	High P, Low DC	32.21	5.07	
	Low P, High DC	31.98	4.55	
	High P, High DC	32.63	4.20	
Gratitude	Low P, Low DC	22.88	2.81	(3,323) = 1.988, *p* = 0.116
	High P, Low DC	22.98	4.50	
	Low P, High DC	23.39	2.44	
	High P, High DC	23.96	3.29	

Note. *SD* Standard deviation, *F* F-statistic, *P* Five precepts, *DC* Death contemplation

## Discussion

This study examined whether elderly Buddhist meditators who strictly adhere to the Five Precepts and regularly engage in death contemplation (maranasati) possess superior psychological well-being, life satisfaction, self-esteem, gratitude, and pāramī-based inner strengths, as well as reduced anxiety, depression, and aggression. Our results confirmed that participants with both strong ethical and contemplative practices demonstrated the most significant psychological benefits. Specifically, this group consistently showed higher inner strengths, greater well-being, higher life satisfaction, and better self-esteem, along with lower anxiety, depression, and aggression compared to all other groups. These findings suggest that integrating ethical conduct and contemplative practice yields psychological benefits beyond either practice alone, supporting the Buddhist perspective that a holistic approach fosters greater resilience and mental health among older adults. Notably, gratitude did not differ significantly between groups, suggesting these practices may have less influence on it. Mean scores for precept observance and meditation frequency in this sample were notably higher than those found in the general older Thai population, supporting the heightened religious commitment of active meditators [14]. Consistent with this, participants manifested higher inner strengths and positive mental health indicators, and lower negative outcomes, compared to national averages.

A major contribution of this study is the nuanced dissection of precept adherence and death contemplation. The four-group analysis demonstrated that high adherence to the Five Precepts was the most consistent and powerful predictor of favorable psychological outcomes, including greater inner strengths, well-being, life satisfaction, and lower levels of anxiety, depression, and aggression. However, the combination of strong ethical conduct and frequent maranasati practice yielded the best results across all domains. In contrast, frequent death contemplation without strong precept adherence (Low Precepts, High Death Contemplation) did not confer significant benefits—underscoring the foundational importance of ethical conduct in Buddhist mental training.

Post hoc analyses further revealed that dedicated practitioners (those with high precept adherence, regardless of maranasati frequency) consistently exhibited significantly greater psychological well-being and inner strengths, and experienced lower levels of negative emotion, than those with lower precept adherence. These results align with previous literature demonstrating the broad protective effects of adherence to Buddhist precepts [14, 59, 60]. Furthermore, our findings suggest that death contemplation provides additional, incremental benefits for resilience and well-being [61, 62]. Existing research suggests mechanisms for these effects: Buddhist practices such as precepts and maranasati foster mindfulness and insight into impermanence [62–64], help reduce rumination and reactivity, and enhance emotional resilience [65]. Loving-kindness (mettā) and self-compassion practices further nurture positive emotion and self-acceptance, which buffer against anxiety and depression [66, 67], while diminished egocentricity and deepened ethical commitment can reduce aggression and promote social harmony [68–70].

These findings extend prior research by demonstrating that combining rigorous adherence to ethical precepts with contemplative wisdom practices yields particularly robust psychological benefits among elderly Buddhist meditators. Importantly, death contemplation alone, in the absence of ethical discipline, was insufficient to produce marked mental health improvements. This synergy echoes traditional Buddhist teachings, which hold virtue (sīla) as the bedrock of meditative and psychological development.

## Limitations

Despite these strengths, several limitations must be acknowledged. First, the use of self-report measures may introduce subjectivity or social desirability bias, particularly concerning desirable qualities such as patience, generosity, and wisdom. Incorporating behavioral observation—such as documented prosocial actions—would strengthen future research.

Second, the sample was limited to Thai Theravāda Buddhist practitioners, which may affect generalizability. Other Buddhist traditions (such as Mahāyāna and Vajrayāna) or secular mindfulness contexts may vary in how they integrate precepts and contemplation of death. Additionally, in cultures where death is a more sensitive or taboo topic, the effects of maranasati practice may differ. Cross-cultural studies are therefore needed to explore these nuances. Lastly, the cross-sectional design of this study limits causal interpretations. Future longitudinal and intervention studies are necessary to determine whether ongoing adherence to precepts and the practice of maranasati leads to sustained psychological growth and well-being.

## Conclusion

This study provides new insights into the mental health benefits linked to advanced Buddhist practice in later life. Elderly practitioners who integrate perfect precept adherence with regular death contemplation exhibit greater inner strengths and superior mental health, including lower aggression, anxiety, and depression, and higher well-being and life satisfaction than those who are less engaged in these practices. These findings highlight the importance of integrating ethical discipline with contemplative wisdom and suggest that spiritual maturation may contribute to resilience and flourishing among aging populations. Future research should expand to include more diverse groups, incorporate behavioral markers, and employ longitudinal designs to elucidate causal pathways better and improve generalizability.

## Data Availability

The datasets used and/or analyzed during the current study are available from the corresponding author upon reasonable request.
